# Genome-wide network-based pathway analysis of CSF t-tau/Aβ1-42 ratio in the ADNI cohort

**DOI:** 10.1186/s12864-017-3798-z

**Published:** 2017-05-30

**Authors:** Wang Cong, Xianglian Meng, Jin Li, Qiushi Zhang, Feng Chen, Wenjie Liu, Ying Wang, Sipu Cheng, Xiaohui Yao, Jingwen Yan, Sungeun Kim, Andrew J. Saykin, Hong Liang, Li Shen, for the Alzheimer’s Disease Neuroimaging Initiative

**Affiliations:** 1 College of Automation, Harbin Engineering University, 145 Nantong Street, BLDG 61-5029, Harbin, 150001 China; 2 Harbin Huade University, No.288 Xue Yuan Rd. Limin Development Zone, Harbin, 150025 China; 3 College of Information Engineering, Northeast Dianli University, 169 Changchun Street, Jilin City, Jilin 132012 China; 4 Department of Radiology and Imaging Sciences, Indiana University School of Medicine, 355 W 16th St, Suite 4100, Indianapolis, IN 46202 USA; 5 School of Informatics and Computing, Indiana University, 719 Indiana Avenue, Indianapolis, IN 46202 USA; 6 Indiana University Network Science Institute, Bloomington, IN 47405 USA

**Keywords:** Alzhermer’s disease, CSF biomarker, t-tau/Aβ_1–42_ ratio, Network analysis, Pathway analysis, Consensus module, CM-iPINBPA

## Abstract

**Background:**

The cerebrospinal fluid (CSF) levels of total tau (t-tau) and Aβ_1–42_ are potential early diagnostic markers for probable Alzheimer’s disease (AD). The influence of genetic variation on these CSF biomarkers has been investigated in candidate or genome-wide association studies (GWAS). However, the investigation of statistically modest associations in GWAS in the context of biological networks is still an under-explored topic in AD studies. The main objective of this study is to gain further biological insights via the integration of statistical gene associations in AD with physical protein interaction networks.

**Results:**

The CSF and genotyping data of 843 study subjects (199 CN, 85 SMC, 239 EMCI, 207 LMCI, 113 AD) from the Alzheimer’s Disease Neuroimaging Initiative (ADNI) were analyzed. PLINK was used to perform GWAS on the t-tau/Aβ_1–42_ ratio using quality controlled genotype data, including 563,980 single nucleotide polymorphisms (SNPs), with age, sex and diagnosis as covariates. Gene-level *p*-values were obtained by VEGAS2. Genes with *p*-value ≤ 0.05 were mapped on to a protein-protein interaction (PPI) network (9,617 nodes, 39,240 edges, from the HPRD Database). We integrated a consensus model strategy into the iPINBPA network analysis framework, and named it as CM-iPINBPA. Four consensus modules (CMs) were discovered by CM-iPINBPA, and were functionally annotated using the pathway analysis tool Enrichr. The intersection of four CMs forms a common subnetwork of 29 genes, including those related to tau phosphorylation (*GSK3B, SUMO1, AKAP5, CALM1* and *DLG4*), amyloid beta production (*CASP8, PIK3R1, PPA1, PARP1, CSNK2A1, NGFR,* and *RHOA*), and AD (*BCL3, CFLAR, SMAD1,* and *HIF1A*).

**Conclusions:**

This study coupled a consensus module (CM) strategy with the iPINBPA network analysis framework, and applied it to the GWAS of CSF t-tau/Aβ1-42 ratio in an AD study. The genome-wide network analysis yielded 4 enriched CMs that share not only genes related to tau phosphorylation or amyloid beta production but also multiple genes enriching several KEGG pathways such as Alzheimer’s disease, colorectal cancer, gliomas, renal cell carcinoma, Huntington’s disease, and others. This study demonstrated that integration of gene-level associations with CMs could yield statistically significant findings to offer valuable biological insights (e.g., functional interaction among the protein products of these genes) and suggest high confidence candidates for subsequent analyses.

## Background

Alzheimer’s disease (AD) is a neurodegenerative disease and the most common form of dementia. Although its etiology is not completely understood, a genetic component of susceptibility to AD has been shown in the literature [1–6]. Cerebrospinal fluid (CSF) studies [7–10] have also been conducted in AD to identify differential biomarkers. Given that one hallmark of AD pathology is a cerebral accumulation of amyloid-β 1–42 peptide (Aβ_1–42_) in amyloid plaques, the Aβ_1–42_ level has been shown markedly reduced in CSF. In addition, the total tau (t-tau) protein level has been shown significantly elevated in the CSF of AD patients. As a result, the CSF t-tau/Aβ_1–42_ ratio has also been studied as a biomarker for differentiating AD from normal older adults [5, 11–13].

With the recent advances in high-throughput genotyping technologies, Genome-Wide Association Studies (GWAS) have been applied to the analysis of CSF biomarkers (e.g., [13, 14]) to identify relevant genetic markers, such as Single Nucleotide Polymorphisms (SNPs). While most studies examined genetic associations with CSF biomarkers at the individual SNP or gene level, mining higher level genetic associations using biological interaction networks is still an under-explored topic for the CSF biomarker studies in AD. Recently, many studies in other domains have demonstrated that integrative analyses of GWAS data and protein-protein interaction (PPI) networks can provide valuable biological insights. Some methods have been proposed to identify subnetworks enriched by GWAS results [15–19]. One tool is iPINBPA [16, 20–23], which is based on the fact that the genes identified in GWAS are more likely to physically interact as well as to belong to the same or related pathways.

With these observations, in this work, we performed a genome-wide network-based pathway analysis for CSF studies in AD. We analyzed an AD cohort from Alzheimer’s Disease Neuroimaging Initiative (ADNI), used the CSF biomarker t-tau/Aβ_1–42_ ratio as the test phenotype or quantitative trait (QT), downloaded the PPI network from the Human Protein Reference Database (HPRD) (http://www.hprd.org/), and applied the iPINBPA analysis to the GWAS findings of the CSF t-tau/Aβ_1–42_ ratio. Our goal was to search for subnetworks or network modules enriched by the CSF GWAS findings, which may offer valuable biological insights and suggest high confidence candidates for subsequent analyses.

## Methods

**Fig. 1:**
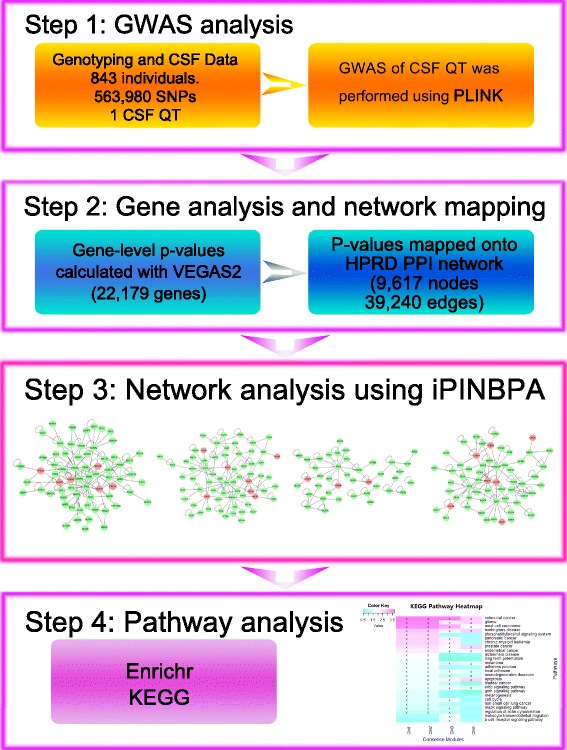
Flow chart. Step1: GWAS using Plink was performed on 843 ADNI participants. Step2: VEGAS2 was used to obtain gene-level p-values, which were mapped onto the HPRD PPI network. Step3: Network-based analysis was performed by iPINBPA software 10 times, and 10 groups of subnetworks were obtained. For the top subnetwork of each result, we computed a consensus module by intersecting this top subnetwork with the most similar subnetworks obtained in all the other nine results. Step4: KEGG pathway enrichment analysis was performed for each consensus module by Enrichr tool.

Figure 1 illustrates the work-flow of this study. The genotyping and CSF data were downloaded from the Alzheimer’s Disease Neuroimaging Initiative (ADNI) database. GWAS of the CSF QT was performed using the PLINK software [24]. This resulted in 563,980 SNPs with associated *p*-values, which were then assigned to 22,179 genes. The gene assignment and gene-based p-values were calculated using the VEGAS2 software [25]. The nominally significant genes (i.e., gene-based *p*-values ≤ 0.05) were mapped onto the HPRD PPI network [26, 27] and analyzed using the iPINBPA method in order to identify the enriched subnetworks. The Enrichr pathway analysis tool [28] was applied to functionally annotate the subnetwork.

### Subjects

Data used in the preparation of this article were obtained from the Alzheimer’s Disease Neuroimaging Initiative (ADNI) database (adni.loni.usc.edu). One goal of ADNI has been to test whether serial magnetic resonance imaging (MRI), positron emission tomography (PET), other biological markers, and clinical and neuropsychological assessment can be combined to measure the progression of mild cognitive impairment (MCI) and early AD. For up-to-date information, see http://adni.loni.usc.edu/. Appropriate Institutional Review Boards approval occurred at each ADNI site and informed consent was obtained from each participant or authorized representative.

In this study, our analyses were concentrated on 843 ADNI subjects whose genotyping data (after quality control described below) and the baseline CSF biomarker data including t-tau and Aβ_1–42_ were both available. This sample included 199 cognitively normal (CN) subjects, 85 subjects with significant memory concern (SMC), 239 subjects with early mild cognitive impairment (EMCI), 207 subjects with late mild cognitive impairment (LMCI), and 113 subjects with Alzheimer’s disease (AD). Table 1 shows the demographic and clinical characteristics of these participants at the baseline, where the characteristics were analyzed with the statistical software IBM SPSS [29] Version 2 for differences across diagnostic groups using one-way analysis of variance (ANOVA) or Chi-square test.

**Table 1:** Selected demographic and clinical characteristics of 843ADNI participants

	CN (*N* = 199)	SMC (*N* = 85)	EMCI (*N* = 239)	LMCI (*N* = 207)	AD (*N* = 113)	*P*-value
Age (years)	74.4 (5.79)	72.0(5.48)	71.4(7.30)	72.4(7.62)	75.2 (8.19)	*p* < 0.001
Women*	96(48%)	50(59%)	102(43%)	83(40%)	45(40%)	0.002*
Education (years)	16.2 (2.82)	16(2.79)	16.2(2.84)	16.4(2.53)	16.4(2.56)	0.764
APOE e4 allele present	47(24%)	31(36%)	99 (41%)	112(54%)	74 (65%)	*p* < 0.001
CDR-SOB	0.04(0.14)	0.08(0.18)	1.27(0.77)	1.65(0.94)	4.53 (1.70)	*p* < 0.001
Mini mental status examination	29.1(1.18)	29.0(1.2)	28.3(1.62)	27.5(1.75)	23.1 (2.05)	*p* < 0.001
Logical memory immediate recall	14.42(3.00)	14.44(3.34)	11.09(2.68)	7.18(3.06)	4.15 (2.70)	*p* < 0.001
Logical memory delayed recall	13.34(3.13)	13.29(3.31)	8.97(1.73)	3.94 (2.7)	1.52 (1.80)	*p* < 0.001
t-tau/Aβ_1-42_Ratio (i.e., QT for GWAS)	0.40 (0.27)	0.37(0.24)	0.50(0.45)	0.70(0.47)	0.98 (0.49)	*p* < 0.001

*AD* Alzheimer’s disease, *ADNI* Alzheimer’s Disease Neuroimaging Initiative, *CDR–SOB* clinical dementia rating–sum of boxes, *CN* cognitively normal, *SMC* significant memory concern, *EMCI* early mild cognitive impairment, *LMCI* late mild cognitive impairment. Number (%) or mean (s.d.) was shown in each entry. *P*-values were assessed due to significant differences between diagnosis groups, which computed using one-way ANOVA (*except for gender using chi-square test)

### CSF Biomarker Measurement as Quantitative Trait

The amyloid-β 1–42 peptide (Aβ_1–42_) and total tau (t-tau) measured in the baseline CSF samples of the participants were downloaded from the ADNI database. The t-tau/Aβ_1–42_ ratio was computed and used as the quantitative trait in the GWAS.

### Genotyping Data and Quality Control

The genotyping data of the participants were collected using either the Illumina 2.5 M array (a byproduct of the ADNI whole genome sequencing sample) or the Illumina OmniQuad array. For the present analyses, single nucleotide polymorphism (SNP) markers that were present on both arrays were included [6, 30, 31].

Quality control (QC) was performed on the ADNI participants using the PLINK v1.07 software [24] (http://zzz.bwh.harvard.edu/plink/), following a similar procedure described in Li et al. [32]. Briefly, SNPs not meeting any of the following criteria were excluded: (1) SNPs available on both 2.5 M array and OmniQuad array, (2) call rate per SNP ≥ 95%; (3) minor allele frequency ≥ 5% (n = 1,845,510 SNPs were excluded based on Criteria 1–3); and (4) Hardy-Weinberg equilibrium test of *p* ≥ 10^−6^ (*n* = 198 SNPs were excluded) using control subjects only. Participants were excluded from the analysis if any of the following criteria were not satisfied: (1) call rate per subject ≥ 95% (no participant was excluded), (2) sex check (1 participant was excluded), (3) identity check for related pairs (8 sibling pairs and 1 sibling triple were identified with PI_HAT >0.5, 1 participant from each family was randomly selected and included in the study).

Population stratification analysis was performed using EIGENSTRAT [33], and confirmed using STRUCTURE [34]. It yielded 89 study participants who did not cluster with the remaining subjects and with the CEU HapMap samples who are primarily of European ancestry (non-Hispanic Caucasians). These 89 participants were excluded from the analysis. After QC, 563,980 SNPs and 843 individuals remained available for the subsequent GWAS, network and pathway analyses.

### SNP-Level and Gene-Level GWAS Analyses

For GWAS, to examine the main effects, linear regression was implemented by PLINK to evaluate the association between each SNP and the t-tau/Aβ_1–42_ ratio. An additive genetic model was tested with covariates, including age, gender, and diagnosis (through five binary dummy variables indicating CN, SMC, EMCI, LMCI, and AD). Then, the SNP-level *p*-values were obtained.

The VEGAS2 software [25] was used to assign 563,980 SNPs to 22,179 autosomal genes according to positions on the UCSC Genome Browser (out of 26,292 in hg19), and to compute gene-level p values. The software applies simulations from the multivariate normal distribution to employ information from a defined subset of markers within a gene as well as take into account linkage disequilibrium between the markers. To save running time, we use a multi-stage approach to adaptively determine the number of simulations per gene: (Stage 1) we run 10^3^ simulations for all the genes; (Stage 2) we run 10^4^ simulations only for genes with Stage 1 empirical *p*-values ≤ 0.1; (Stage 3) we run 10^6^ simulations only for genes with Stage 2 empirical *p*-values ≤ 0.001. We interpret an empirical *p*-value of 0 from 10^6^ simulations as *p* < 10^−6^. Given 22,179 genes included in this analysis, a Bonferroni-corrected threshold is *p* < 2.25 × 10^−6^ (i.e., 0.05/22,179), which can be exceeded by the theoretically smallest empirical *p*-value shown above. A Manhattan plot was generated using R (http://www.r-project.org) to visualize the gene-level GWAS results for our work.

### Network-level Analysis

The Human PPI data (*n* = 9,617) were downloaded from the Human Protein Reference Database (HPRD, http://www.hprd.org); gene-level *p*-values obtained from the GWAS of the CSF t-tau/Aβ_1–42_ ratio were mapped to the PPI network. The integrative protein interaction network-based pathway analysis (iPINBPA) software [22] was used to integrate GWAS findings with physical evidence of interaction at the protein level, and to detect new high-level associations (i.e., subnetworks of functionally interacted genes) with the CSF biomarker. Briefly, iPINBPA identifies enriched subnetworks using the following three steps.

In Step 1, using the GWAS findings, the nominally significant genes (i.e., *p* ≤ 0.05) are treated as seed genes, and assigned with certain weights (e.g., in this work, *1* for seed genes, *0* for the rest). After that, a random walk with restart strategy is employed to smooth these weights over the entire network. Intuitively, the nodes in the network are weighted based on their connectivity to seed genes (i.e., guilt-by-association). Let n_k_ be a node on the PPI network mapped with gene-level *p*-value p_i_. Let e_ij_ be the edge connecting n_i_ and n_j_, and W_ij_ be the weight of e_ij_. All the W_ij_’s form the adjacency matrix W. Extending Köhler’s approach [35], iPINBPA weights the edge e_ij_ as follows:

$$ {W}_{i j} = \left(\left(1-{p}_i\right) + \left(1-{p}_j\right)\right)/2. $$

In addition, it normalizes the adjacency matrix W by its columns. After each step of random walk, a score vector is calculated as

$$ P(t) = \left(1- r\right) W \cdot p\ P\left( t-1\right) + r P(0), $$

where P(t) is the score after walking t steps, and r is the restart ratio. In this work, we assign *1* to all the seed genes, and *0* to the rest. Upon the completion of the random walk after *T* steps, the vector *P(T)* contains the node weights, which reflect the topological connections to the seed genes [36].

In Step 2, given a network *A* containing *k* nodes, iPINBPA defines its score by combining the gene-level *p*-values with node weights described above, using the Liptak-Stouffer method. Specifically, the network score of *A* is defined, via weighted Z transform test [37], as follows:

$$ {Z}_A=\frac{{\displaystyle {\sum}_{i\in A} P{(T)}_i{z}_i}}{\sqrt{{\displaystyle {\sum}_{i\in A} P{(T)}_i^2}}} $$.

A random sampling of gene sets of size *k*∈[1, 500] for 1000 times was applied in iPINBPA [36] to determine the background distribution of the network score. Using this distribution, the adjusted network score of *A* is defined as:

$$ {S}_A=\frac{Z_A-{\mu}_k}{\sigma_k} $$,

where Z_A_ is the network score, and *μ*_*k*_ and *σ*_*k*_ are respectively the mean and the standard deviation of the background distribution of the network scores at size *k*.

In Step 3, a greedy algorithm was developed to search for modules with high network scores, i.e., those enriched in genes with low *p*-values and high weights. Details about the algorithm is available in Wang et al. [22].

In this work, the parameters were set in iPINBPA as follows: r = 0.5, T = 5, NetScore > 3.0, NetSize ≥ 5, and MaxNetSize ≤ 300. Given the stochastic nature of the iPINBPA algorithm, we ran iPINBPA ten times, respectively by setting the random seed value from 1 to 10. Consequently, we obtained ten groups of enriched subnetworks (GNs) identified by iPINBPA. Below, we couple a consensus module (CM) strategy with iPINBPA (named as CM-iPINBPA) to generate consensus findings from these analyses.

Given two subnetworks *m* and *n*, we use Dice’s coefficient *DC(m,n)* to measure their similarity:

$$ D C\left( m, n\right)=\frac{2\left| m\cap \right.\left. n\right|}{\left| m\right|+\left| n\right|} $$.

In this work, we only focused on analyzing the top subnetwork (TN) in each iPINBPA run. Let *TN*_*a*_ be the top subnetwork identified in Run *a* ∈ {1, 2, …, 10}. We first find *SN*_*b*_ *(TN*_*a*_ *)*, which is the most similar subnetwork to *TN*_*a*_ in Run *b* ∈ {1, 2, …, 10}\{*a*}. Clearly we have

$$ S{N}_b\left( T{N}_a\right) = argma{x}_{sn} D C\left( T{N}_a,\  sn\right), $$

where *sn* is any subnetwork enriched in Run *b*. After that, we define the consensus module (CM) based on Run *a* as follows:

*CM*_*a*_ = *TN*_*a*_ ∩ (∩_*a* ≠ *b*,*b* ∈ {1,2,…,10}_ *SN*_*b*_(*TN*_*a*_)).

Namely, *CM*_*a*_ is the intersection of *TN*_*a*_ and its most similar subnetworks identified in all the other runs.

### Network Module Visualization and Functional Analysis

Cytoscape 3.2 [38] was used to visualize the example identified network modules. The Enrichr [28] pathway analysis tool (http://amp.pharm.mssm.edu/Enrichr/) and the Kyoto Encyclopedia of Genes and Genomes database (KEGG; http://www.genome.jp/kegg/) [39] was applied to functional analysis of the identified network modules. Heat map was plotted, using R 3.2.0 software, to demonstrate relations between consensus modules and relevant KEGG pathways.

## Results

### GWAS and gene-level analysis

The demographic and clinical characteristics for the 843 ADNI subjects in this study are presented in Table 1. The summary statistics for all diagnostic groups (CN, SMC, EMCI, LMCI, and AD) are given. Education level (*p* = 0.764) was not significantly different across the five diagnostic groups; however, gender demonstrated a significant difference (*p* = 0.002). Furthermore, as expected, age, *APOE* e4 status, clinical dementia rating–sum of boxes (CDR-SOB), mini mental status examination (MMSE), logical memory immediate recall, and logical memory delayed recall exhibited significant differences across the five groups (*p* < 0.001). Also as expected, the phenotype t-tau/Aβ_1–42_ ratio significantly differed across the diagnostic groups (*p* < 0.001).

The top SNP in the GWAS analysis was rs4420638 (chromosome 19, 14 kb away from the *APOC1* gene, *p* = 2.576E-28), which was previously reported by Lars Bertram et al. [40]. The SNP rs769449 within the *APOE* gene on chromosome 19 was also significant with *p* = 4.98E-23, and was previously reported by Soerensen et al. [41]. Similar to the results reported in our earlier paper [13], The *TOMM40* SNP rs2075650 (chromosome 19, *p* =4.23E-18, was associated with t-tau/Aβ_1–42_ ratio.

**Fig. 2:**
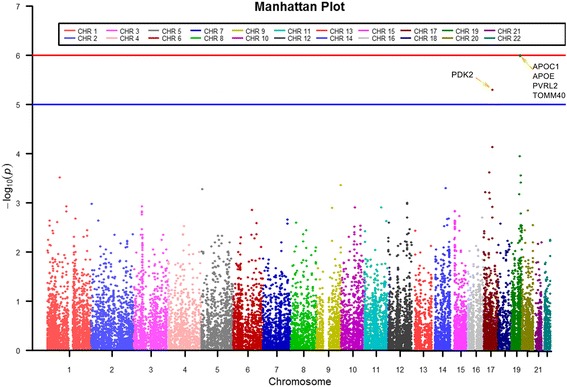
**a** Manhattan plot showing the gene-level p values in ADNI t-tau/Aβ_1–42_ ratio GWAS study. The blue line corresponds to *p* = 10^−5^; the red line corresponds to *p* = 10^−6^. Bonferroni-corrected threshold is p < 2.25 × 10^−6^ (i.e., 0.05/22,179)

Under the hypothesis that genes, rather than SNPs, are the functional units in biology [42], a gene-level association analysis of the t-tau/Aβ_1–42_ ratio was performed based on the SNP-level results by VEGAS2. Table 2 shows the top 10 genes identified by VEGAS2. Figure 2 shows the Manhattan plot of the gene-based GWAS results.

**Table 2:** The top 10 genes identified by VEGAS2

Chr	Gene	nSNPs	Test	*P*value	TopSNP	TopSNP-*P*value
19	APOC1	6	274.96	1.00E-06	rs4420638	2.58E-28
19	APOE	7	188.92	1.00E-06	rs769449	4.98E-23
19	PVRL2	19	228.01	1.00E-06	rs2075650	4.23E-18
19	TOMM40	11	363.85	1.00E-06	rs769449	4.98E-23
17	PDK2	11	103.37	5.00E-06	rs3809762	1.86E-06
17	ITGA3	15	111.81	7.40E-05	rs3809762	1.86E-06
19	CBLC	2	24.35	1.12E-04	rs2965121	1.45E-04
17	CCL7	5	28.84	2.38E-04	rs991804	2.28E-04
19	KLK7	12	56.02	2.76E-04	rs11084043	7.74E-05
1	PTGER3	97	400.30	3.07E-04	rs7540868	1.89E-04

Chr: Chromosome; Gene: Gene name; nSNPs: Number of SNPs in the input file that map to the gene; Test: The sum of the individual chi-squared 1 degree of freedom SNP-association test statistic; Pvalue: The gene-based p-value considering the full set of SNPs; TopSNP: The name of the most significant SNP within the gene; TopSNP-Pvalue: The p-value for the most significant SNP with the gene

### Network search for CMs

Consensus modules (CMs) were identified by CM-iPINBPA network analysis strategy. Subnetwork search was conducted on the GWAS findings using iPINBPA ten times by varying the seed value of random number generator, which ranged from 1 to 10. Table 3 summarizes the results of ten iPINBPA runs. *PRKCA* and *TP53* appeared to be the start nodes of the top subnetworks identified in multiple runs. The *PRKCA* gene was previously reported as being associated with an altered amyloid precursor protein (APP) secretion in fibroblasts from AD patients [43, 44]. Culmsee et al. demonstrated that *TP53* was a novel gene as a biomarker of AD and was related to neurodegenerative processes [45].

**Table 3:** Results of 10 iPINBPA runs

SRN	# of Subnetworks	TN Size	TNStartNode	TN Score
1	1058	99	PRKCA	10.11
2	918	92	PRKCA	9.70
3	975	151	TP53	10.95
4	1100	133	-	10.52
5	977	108	PRKCA	9.85
6	936	135	-	10.64
7	1166	101	PRKCA	9.41
8	1050	147	-	11.27
9	972	67	TP53	8.04
10	955	146	-	11.25

SRN:The seed value used for an iPINBPA run

# of Subnetworks: The number of subnetworks identified in an iPINBPA run

TN Size: The number of genes in the top subnetwork identified in an iPINBPA run

TN StartNode: The start node of the top subnetwork

TN Score: The score of the top subnetwork

**Fig. 3:**
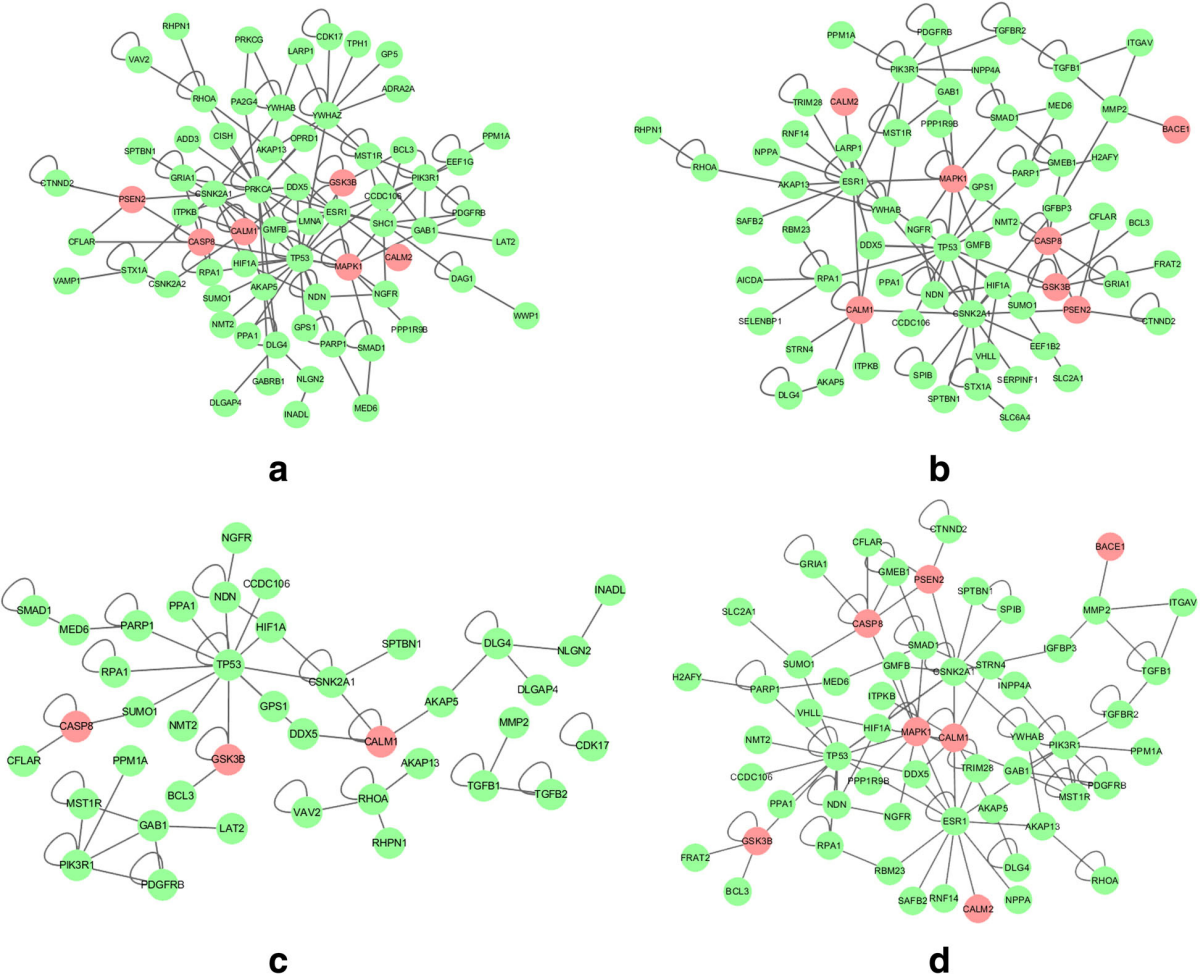
Consensus modules identified by 10 runs of iPINBPA. **a** Consensus Module1; **b** Consensus Module 2; (c) Consensus Module 3; (d) Consensus Module 4. The reddish color indicates genes belonging to the KEGG Alzheimer’s Pathway. The adjusted network scores (i.e., *S*_*A*_) of these four modules are 10.15, 10.39, 7.99, and 10.46, respectively. Therefore, all the modules are significant (*p* < 0.001)

Table 4 shows the top subnetwork identified in each run, its most similar subnetworks in other runs coupled with the Dice’s coefficient value, and the corresponding CM. For example, in Table 4, the Dice’s coefficient between TN1 and SN1 (in GN_2_) is 0.96335. Thanks to the overlapping subnetworks, only four unique CMs were identified (Table 4). These four CMs are shown in Fig. 3, where the reddish nodes represent the known AD genes from the KEGG AD pathway (hsa05010). CM1 (*S*_*A*_ = 10.15, p < 0.001) shown in Fig. 3(a) contains totally 67 genes, including KEGG AD genes *GSK3B, MAPK1, PSEN2, CALM1, CALM2* and *CASP8*. CM2 (*S*_*A*_ = 10.39, p < 0.001) shown in Fig. 3(b) contains totally 67 genes, including KEGG AD genes *BACE1, GSK3B, MAPK1, PSEN2, CALM1, CALM2* and *CASP8*. CM3 (*S*_*A*_ = 7.99, *p* < 0.001) shown in Fig. 3(c) contains 40 genes, including KEGG AD genes *GSK3B*, *CALM1* and *CASP8*. CM4 (*S*_*A*_ = 10.46, *p* < 0.001) shown in Fig. 3(d) contains 58 genes, including KEGG AD genes *BACE1*, *GSK3B*, *MAPK1*, *PSEN2*, *CALM1*, *CALM2* and *CASP8*.

**Table 4:** The characteristics of the identified consensus modules in 10 iPINBPA runs

CM	RunA	Ta: the top subnetwork in RunA. Sb: the most similar subnetwork to Ta in RunB										
		RunB	1	2	3	4	5	6	7	8	9	10
1	1	Rank of Sb in RunB	1	1	4	3	1	6	1	3	8	4
		DC(Ta, Sb)	1.00	0.96	0.88	0.88	0.96	0.96	0.99	0.82	0.81	0.96
1	2	Rank of Sb in RunB	1	1	4	3	1	6	1	3	8	4
		DC(Ta, Sb)	0.96	1.00	0.85	0.85	0.92	1.00	0.95	0.78	0.84	0.92
2	3	Rank of Sb in RunB	3	2	1	2	3	2	3	2	1	5
		DC(Ta, Sb)	0.78	0.77	1.00	0.93	0.83	0.91	0.81	0.97	0.61	0.83
3	4	Rank of Sb in RunB	2	3	2	1	2	1	2	1	13	1
		DC(Ta, Sb)	0.85	0.82	0.97	1.00	0.89	0.99	0.86	0.95	0.67	0.95
1	5	Rank of Sb in RunB	1	1	4	3	1	6	1	3	8	4
		DC(Ta, Sb)	0.96	0.92	0.93	0.93	1.00	0.92	0.97	0.86	0.77	1.00
3	6	Rank of Sb in RunB	2	3	2	1	2	1	2	1	13	1
		DC(Ta, Sb)	0.84	0.82	0.98	0.99	0.88	1.00	0.86	0.96	0.66	0.96
1	7	Rank of Sb in RunB	1	1	4	3	1	6	1	3	8	4
		DC(Ta, Sb)	0.99	0.95	0.89	0.89	0.97	0.95	1.00	0.83	0.80	0.97
3	8	Rank of Sb in RunB	2	3	2	1	2	1	2	1	13	1
		DC(Ta, Sb)	0.80	0.78	0.98	0.95	0.84	0.96	0.81	1.00	0.63	1.00
4	9	Rank of Sb in RunB	3	2	1	2	3	8	3	2	1	5
		DC(Ta, Sb)	0.82	0.83	0.61	0.68	0.77	0.73	0.79	0.64	1.00	0.77
3	10	Rank of Sb in RunB	2	3	2	1	2	1	2	1	13	1
		DC(Ta, Sb)	0.80	0.78	0.98	0.95	0.85	0.96	0.82	1.00	0.63	1.00

*CM* consensus module id, *DC* Dice’s coefficient

In addition, the intersection of the four CMs was extracted and named as the common subnetwork. The common subnetwork is shown in Fig. 5, and contains total 29 genes, including 3 KEGG AD genes *CALM1, CASP8,* and *GSK3B,* and 26 other genes.

### Pathway analysis of consensus modules and the common subnetwork

**Fig. 4:**
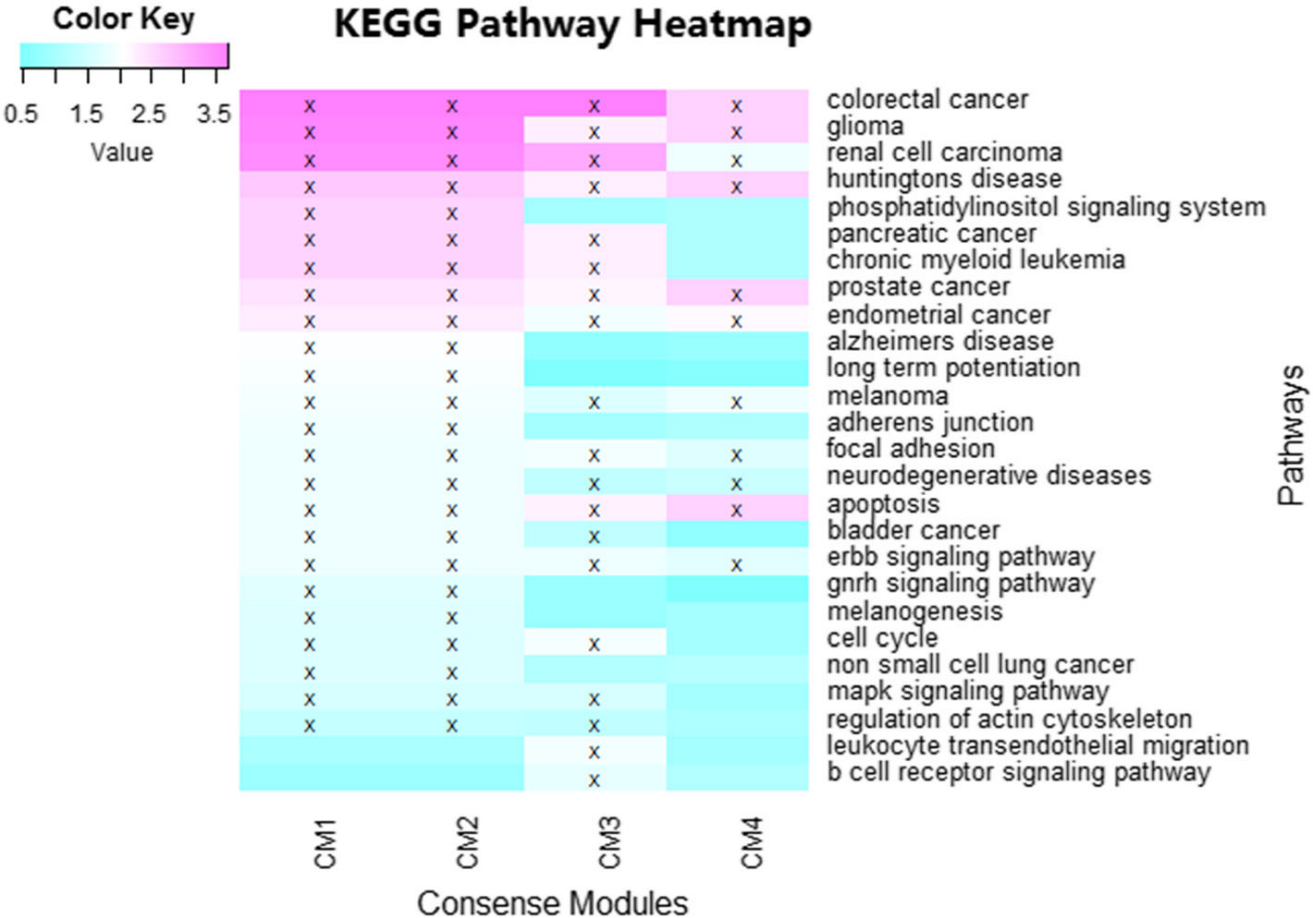
Functional annotation of the four identified consensus modules (CM1-CM4) using KEGG pathways. The four consensus modules were treated as four gene sets, and went through pathway enrichment analysis based on the KEGG pathway database. The enrichment results at a nominal statistical threshold of adjusted *p*-value < 0.05 are shown. -log_10_(p-value) values are color-mapped and displayed in the heat map. Heat map blocks labeled with “x” reach the nominal significance level of adjusted *p*-value < 0.05. Only top enrichment findings are included in the heat map, and so each row (pathway) has at least one “x” block

To test the hypothesis that CMs enriched by the GWAS findings might be significantly overrepresented in AD and other relevant pathways, the Enrichr method was performed for pathway analysis. For the genes in each CM, a pathway enrichment analysis was conducted, and the nominally enriched pathways were identified with adjusted *p*-value ≤ 0.05. Then, these identified pathways of the four CMs were plotted as a heat map shown in Fig. 4 to summarize the relationships between the pathways and CMs. Note that Fig. 4 lists only pathways enriched by at least one CM. Table 5 shows top 20 pathway enrichments analysis of the common subnetwork.

**Table 5:** Top 20 pathway enrichments analysis of the common consensus subnetwork

Pathway	Overlap	*P*-value	Adjusted *P*-value	Z-score	Combined Score	Genes
glioma	4/62	7.19E-05	0.002277	−2.14	13.00	PDGFRB; PIK3R1;CALM1;TP53
apoptosis	4/81	1.94E-04	0.002277	−1.85	11.28	CASP8; CFLAR;PIK3R1;TP53
huntingtons disease	3/31	2.20E-04	0.002277	−1.77	10.77	CASP8;CALM1;TP53
colorectal cancer	4/84	2.22E-04	0.002277	−1.84	11.18	PDGFRB; GSK3B;PIK3R1;TP53
prostate cancer	4/86	2.42E-04	0.002277	−1.80	10.97	PDGFRB; GSK3B;PIK3R1;TP53
endometrial cancer	3/52	9.23E-04	0.007232	−1.65	8.13	GSK3B;PIK3R1;TP53
renal cell carcinoma	3/69	2.02E-03	0.01286	−1.61	6.99	GAB1;PIK3R1;HIF1A
melanoma	3/71	2.19E-03	0.01286	−1.49	6.50	PDGFRB;PIK3R1;TP53
erbb signaling pathway	3/85	3.59E-03	0.018769	−1.53	6.08	GSK3B;GAB1;PIK3R1
focal adhesion	4/192	4.51E-03	0.02118	−1.60	6.16	PDGFRB; GSK3B;PIK3R1;RHOA
neurodegenerative diseases	2/38	9.01E-03	0.038512	−0.87	2.83	NGFR;CASP8
non small cell lung cancer	2/53	1.66E-02	0.064347	−1.21	3.32	PIK3R1;TP53
basal cell carcinoma	2/55	1.78E-02	0.064347	−0.92	2.53	GSK3B;TP53
b cell receptor signaling pathway	2/62	2.22E-02	0.074377	−0.91	2.37	GSK3B;PIK3R1
phosphatidylinositol signaling system	2/72	2.91E-02	0.081711	−1.00	2.50	PIK3R1;CALM1
pancreatic cancer	2/73	2.98E-02	0.081711	−0.99	2.49	PIK3R1;TP53
chronic myeloid leukemia	2/75	3.13E-02	0.081711	−0.93	2.33	PIK3R1;TP53
adherens junction	2/75	3.13E-02	0.081711	−0.78	1.96	CSNK2A1;RHOA
regulation of actin cytoskeleton	3/201	3.54E-02	0.087678	−0.96	2.34	PDGFRB; PIK3R1;RHOA
small cell lung cancer	2/85	3.92E-02	0.092081	−0.73	1.74	PIK3R1;TP53

Pathway: The name of KEGG pathway

Overlap: The number of overlapping genes compared with the number of input genes

*P*-value: *P*-value was computed using the Fisher exact test

Adjusted P-value: Adjusted *P*-value was a corrected *p*-value to the Fisher exact test

Z-score: Computed by assessing the deviation from the expected rank

Combined score: Computed by taking the log of the *p*-value from the Fish exact test and multiplying that by the z-score of the deviation from the expected rank

Genes: The overlapping genes between the input and the pathway

## Discussion

### Gene-level analysis

In the GWAS analysis, the gene-level *p*-values were determined and shown in Fig. 2. The use of the CSF t-tau/Aβ_1–42_ ratio as a quantitative trait (QT) in this study enabled us to examine the effect of genes previously known to be associated with the QT as well as to identify novel genes. Table 2 lists the top ten genes obtained by converting SNPs into gene-wise *p*-values. Given the Bonferroni-corrected threshold of *p* < 2.25 × 10^−6^ (i.e., 0.05/22,179), we found five significant genes. As expected, significant associations were identified between loci on chromosome 19 and the CSF t-tau/Aβ_1–42_ ratio (e.g., *APOC1, APOE, PVRL2, TOMM40*, *p* = 1 × 10^−6^, see Fig. 2). Among these genes, apolipoprotein C1 (ApoC1) encoded by the *APOC1* gene is associated with amyloid β plaques; the *APOE* and *TOMM40* (rs769449) genes code for proteins related to the clearance of Aβ and mitochondrial functions [5, 13]; and the *PVRL2* gene was previously reported as related to risk factors that contribute to AD pathogenesis [46]. *PDK2* (*p* = 5 × 10^−6^) shows a trend towards the significance, and the overexpression of this gene may be related to cancer and diabetes[47]. Additionally, *CCL7* mRNA is highly increased by Aβ_1–42_ stimulation [48]. The *CCL7* gene was previously reported as related to the chemotaxis of pro-inflammatory cells to the inflamed location [49]. The *PTGER3* gene was previously reported as being related to the inflammatory response [50].

### Network search for CMs and functional validation

Although iPINBPA has been successfully applied in several previous studies [16, 20–23], we observed that different subnetworks could be obtained by using different random seed values. To overcome this limitation, we proposed to examine the consensus modules discovered by multiple iPINBPA analyses. In other words, we focused on examining the shared subnetworks among multiple iPINBPA runs, which turned out to be more stable patterns. A two-stage strategy was employed. First, ten groups of subnetworks were generated by running iPINBPA ten times with varying random seed values ranging from 1 to 10. After comparing these ten sets of results, we identified ten CMs, one from each run (defined as the intersection of the top subnetwork from the current run and the most similar subnetworks from all the other runs). As a result, there are four unique CMs based on ten identified ones.

The genes in the CMs might not show a direct statistical significance but could interact with some genes identified in our GWAS. These genes can demonstrate indirect association with the studied QT, and may potentially provide valuable biological interpretation. For example, Consensus Module 1 contains the protein gamma-aminobutyric acid (GABA) A receptor (*GABRB1*) gene. *GABRB1* codes for the β1 subunit of gamma-aminobutyric acid A (GABA_A_) receptors [1]. The *GABRB1* gene has been demonstrated to be involved in the thalamus structure and its interactive effects on intelligence [51]. The *GABRB1* gene had also been associated with many neuropsychological diseases, such as schizophrenia, major depression, bipolar disorder, and Alzheimer’s disease [52].

In this study, we hypothesize that trait prioritized CM with high replication might have strong functional associations with t-tau/Aβ_1–42_ ratio. We gathered these identified pathways for 4 CMs to plot heatmap to explore and refine the relationships between pathways and CMs (Fig. 4). In Fig. 4, it was observed that four pathways, including colorectal cancer, gliomas, renal cell carcinoma, and Huntington’s disease, were commonly enriched in all the consensus modules. The neurodegenerative symptoms of neuron death affect many diseases, including Alzheimer’s, Parkinson’s, and Huntington’s diseases. Below we briefly discuss a few additional pathways identified in Fig. 4. In AD, focal adhesion complexes regulating neuronal viability can be used in treatment to adjust neuronal survival [53]. The adherens junction has been demonstrated as maintaining blood–brain barrier integrity, and the adherens junction pathways is highly associated with neurodegenerative diseases [54]. Apoptosis is an important pathway in Alzheimer’s disease that is associated with neuronal loss [55]. The change in the MAPK signaling pathway contributes to significant change in neurotropin [56]. Some cancer-related pathways were found, such as colorectal cancer, pancreatic cancer, prostate cancer, endometrial cancer, bladder cancer, and so on. Some prior studies have been performed to examine the relationship between cancer and neurologic disease [57, 58].

With these observations, the genes in the CMs may provide valuable information to suggest novel molecular mechanisms for subsequent analyses. Compared with the standard iPINBPA method, CM-iPINBPA network analysis strategy for mining consensus models could offer more stable results.

### Common subnetwork and functional validation

**Fig. 5:**
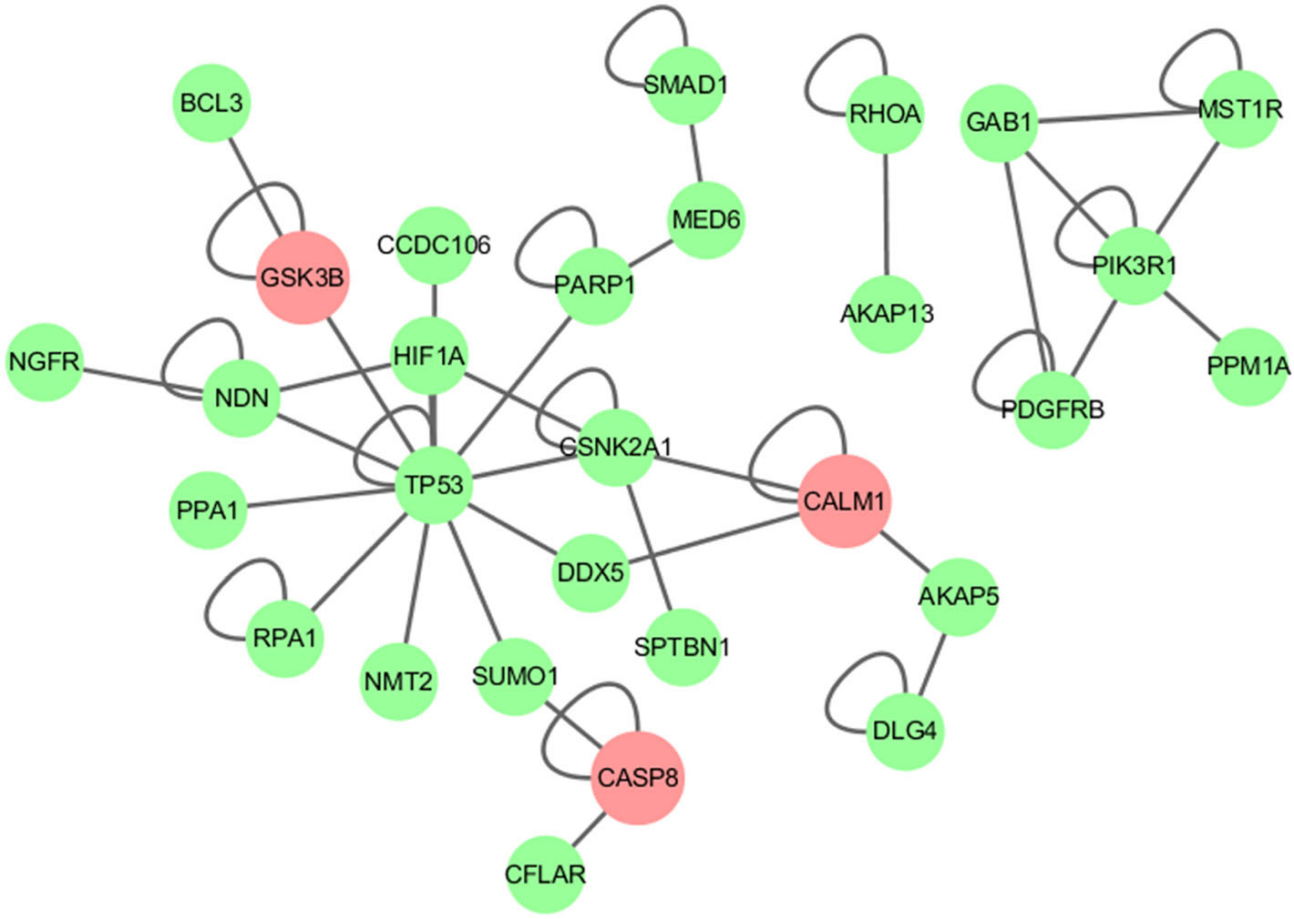
The common subnetwork. This subnetwork consists of only overlapping genes of all consensus modules. The reddish color indicates genes belonging to the KEGG Alzheimer’s Pathway

The common subnetwork is the intersection of the four identified common modules, and consists of 29 genes (Fig. 5). Among these genes, the *GSK3B, SUMO1, AKAP5, CALM1* and *DLG4* genes have been identified to be involved in tau phosphorylation, and over-phosphorylation of the tau protein can form the tangles in the brain of AD patients [59–63]. Additionally, the *CASP8, PIK3R1, PPA1, PARP1, CSNK2A1, NGFR,* and *RHOA* genes have been demonstrated to be involved in amyloid beta peptide production [64–70]. The *BCL3, CFLAR, SMAD1,* and *HIF1A* genes have been identified to be associated with late-onset Alzheimer's disease. The common subnetwork also contains the following genes *TP53, DDX5, NDN, MST1R, CCDC106, NMT2, RPA1, AKAP13, GAB1, PPM1A, SPTBN1* and *MED6*, which interact with themselves and other genes. These findings offer valuable biological insights and suggest promising candidates for subsequent analyses.

Table 5 shows the top twenty pathways enriched by the common subnetwork. Some significant pathways were observed, such as glioma, apoptosis, Huntington’s disease, renal cell carcinoma, melanoma, the erbb signaling pathway, focal adhesion, neurodegenerative diseases, and so on. Twelve genes (*PDGFRB, PIK3R1, CALM1, CFLAR, TP53, RHOA, CASP8, HIF1A, GSK3B, GAB1, NGFR* and *CSNK2A1*) were involved in the top twenty pathways. The gene *PDGFRB* has been confirmed as causative of primary familial brain calcifications (PFBC) [71]. The gene *PIK3R1* has been shown to be involved in Alzheimer’s disease [72]. *CALM1* encodes for tau protein and regulates the subcellular localization and function of calmodulin in neurons [73]. *CFLAR* suppresses death receptor-induced apoptosis and TCR activation, which induces cell death by inhibiting caspase-8 activation [74]. *RHOA* was implicated in Aβ neurotoxicity, and the activation generates cytoskeletal changes [75]. *NGFR* ligands play an important role in preventing fundamental tau-related pathologic mechanisms in Alzheimer’s disease [76]. These pathways appear relevant given the results in prior studies. For other genes identified in this pathway analysis, it warrants further investigation to demonstrate the role they play.

### Limitations

Due to the limited number of subjects available to us, we were only able to perform a discovery study in this work. When more data become available in the future, replication studies in independent cohorts are required to evaluate and validate the identified network modules in our study. In addition, in this work, we reported the results using the default parameter setting provided by the software tool and according to Lili et al. [22], except the random seed value. We ran iPINBPA multiple times by using different random seed values and then extracting the consensus patterns to stabilize the results. As to other parameters, we also briefly tested each of those by varying its value. For most of these parameters, we obtained very similar results. The most sensitive parameter is the restart ratio used in the step called “random walk with restart” for prioritizing phenotype-associated genes. It is excepted that different restart ratios will assign different weights to network nodes and subsequently produce different scores for network components. The determination of the optimal restart ratio warrants a separate and focused investigation and is an interesting future direction.

## Conclusions

Network-based methods form a new generation of enrichment analysis strategy, and they can overcome the limitation of traditional enrichment analysis where only a fixed set of pre-defined pathways are examined. In this study, a genome-wide network-based pathway analysis of the CSF biomarker of the t-tau/Aβ_1–42_ ratio was performed, using a sample of 843 subjects from the ADNI database. To our knowledge, this is the first genome-wide network-based pathway study on the CSF biomarker t-tau/Aβ_1–42_ ratio in Alzheimer’s disease. Due to the stochastic nature of the iPINBPA method, we employed a consensus module (CM) strategy to run iPINBPA multiple times and aimed to identify CMs from these different runs. We identified 4 CMs. These CMs contain not only genes from KEGG AD pathways, including *BACE1, GSK3B, MAPK1, PSEN2, CALM1, CALM2, CASP8,* and *SK3B*; but also interesting genes with relevant biological functions such as *GABRB1*, *MMP2*, *CDK17*, and *IGFBP3*. In sum, besides confirming previous findings (e.g., *APOE*, *TOMM40*, *APOC1*), this study has also suggested new susceptible genes, CMs and pathways underlying Alzheimer’s disease.

## Abbreviations

AD: Alzheimer’s disease
ADNI: Alzheimer’s Disease Neuroimaging Initiative
ANOVA: Analysis of variance
Aβ_1–42_: Amyloid-β 1–42 peptide
CDR-SOB: Clinical dementia rating–sum of boxes
CM: Consensus module
CM-iPINBPA: Consensus module (CM) strategy with iPINBPA
CMs: Consensus modules
CN: Cognitively normal
CSF: Cerebrospinal fluid
DC: Dice’s coefficient
EMCI: Early mild cognitive impairment
GNs: Groups of enriched subnetworks
GWAS: Genome-Wide Association Studies
HPRD: Human Protein Reference Database
iPINBPA: Integrative protein interaction network-based pathway analysis
LMCI: Late mild cognitive impairment
MCI: Mild cognitive impairmen
MMSE: Mini mental status examination.
MRI: Magnetic resonance imaging
PET: Positron emission tomography
PPI: Protein-protein interaction
QT: Quantitative trait
SMC: Significant memory concern
SNPs: Single Nucleotide Polymorphisms
TN: Top subnetwork
t-tau: Total tau
